# MAXILLOFACIAL TRAUMA, ETIOLOGY AND PROFILE OF PATIENTS: AN EXPLORATORY STUDY

**DOI:** 10.1590/1413-785220172506152670

**Published:** 2017

**Authors:** ILKY POLLANSKY SILVA E FARIAS, ÍTALO DE MACEDO BERNARDINO, LORENA MARQUES DA NÓBREGA, RAFAEL GROTTA GREMPEL, SÉRGIO D’AVILA

**Affiliations:** 1. Program in Maxillofacial Surgery and Traumatology, Hospital Regional do Agreste, Caruaru, PE, Brazil.; 2. Department of Dentistry, Universidade Estadual da Paraíba, Campina Grande, PB, Brazil.; 3. Post-graduate Program in Dentistry, Universidade Estadual da Paraíba, Campina Grande, PB, Brazil.; 4. Strategic Technology Center, Universidade Estadual da Paraíba, Campina Grande, PB, Brazil.

**Keywords:** Facial injuries, Facial bones, Traumatology., Traumatismos faciais, Ossos faciais, Traumatologia.

## Abstract

**Objective:**

To describe the profile of patients with facial trauma admitted in a hospital located in a metropolitan area of Northeast Brazil.

**Methods:**

A cross-sectional and exploratory study was performed. A total of 244 cases were in agreement with the eligibility criteria. The variables include the sociodemographic characteristics of patients, etiology, type of trauma, treatment modalities, length of stay in a hospital and quarter of care. Descriptive statistics and Cluster Analysis were performed.

**Results:**

The average age of patients was 31.16 years (SD = 15.17 years) and average hospitalization was 6.32 days (SD = 7.75 days). It was verified the automatic formation of four clusters with different profiles of patients. The variables which most contributed to the external differentiation between clusters were: length of stay in a hospital (p <0.001), etiology (p <0.001), type of facial trauma (p <0.001), presence of associated trauma (p <0.001), treatment modalities (p <0.001) and quarter of care (p <0.001).

**Conclusion:**

The most of patients were men, victims of traffic accidents, which suffered fracture of zygomatic complex and underwent surgery. **Level of Evidence III, Retrospective Study.**

## RESUMO

**Objetivo:**

Traçar o perfil dos pacientes internados com trauma de face em um hospital localizado em região metropolitana do Nordeste do Brasil.

**Métodos:**

Tratou-se de estudo transversal e exploratório. Um total de 244 casos atendeu aos critérios de elegibilidade, sendo incluídos na amostra. As variáveis estudadas incluíram características sociodemográficas das vítimas, etiologia, tipos de traumas, modalidades de tratamento, tempo de internação e trimestre de atendimento. Foi feita estatística descritiva e análise de cluster.

**Resultados:**

A média de idade dos pacientes foi 31,16 anos (DP = 15,17 anos) e o tempo médio de internação foi de 6,32 dias (DP = 7,75 dias). Verificou-se a formação automática de quatro clusters com perfis distintos de pacientes. As variáveis que mais contribuíram para a diferenciação externa entre os clusters foram: tempo de internação (p < 0,001), etiologia (p < 0,001), tipo do trauma facial (p < 0,001), presença de trauma associado (p < 0,001), tipo de tratamento (p < 0,001) e trimestre de atendimento (p < 0,001).

**Conclusão:**

A maioria dos pacientes eram homens, vítimas de acidentes de trânsito, que apresentaram fratura do complexo zigomático, submetidos a tratamento cirúrgico. **Nível de Evidência III, Estudo Retrospectivo.**

## INTRODUCTION

Trauma from external causes represents one of the greatest challenges for public health services in different regions of the world.^1^ ^-^ ^3^ In Brazil, thousands of people are daily victims of interpersonal violence and are involved in traffic accidents, overloading health services and generating high emotional and social costs. Trauma in head, neck and face is one of the most prevalent and among the etiological agents of facial trauma, traffic accidents, falls, aggressions and penetrating wounds (caused by firearms) stand out, with sociodemographic, cultural and environmental factors playing an important role in the epidemiology of these outcomes.^4^ ^-^ ^6^

Depending on severity, the treatment of trauma patients requires multidisciplinary and integrated care. In addition, facial trauma may be accompanied by other types of serious injury, which may result in emotional and psychological problems requiring lifelong follow-up.^7^ ^-^ ^9^ Epidemiological studies are necessary for a better understanding of the distribution patterns of lesions, etiological factors, and for providing valuable information for the planning of health actions.

Understanding the patterns of facial injuries and the victims’ profile may also help managers to refocus and improve the services offered. In this context, this study had the aim of determining the profile of hospitalized patients with facial trauma and describing the characteristics of lesions in an emergency and trauma hospital in a medium-sized city in northeastern Brazil.

## MATERIAL AND METHODS

This was a cross-sectional and exploratory study carried out in a reference hospital in emergency and trauma care located in the city of Campina Grande, Paraíba, Brazil, during the period from January to December 2011. The municipality, which has population estimated at 386,000 inhabitants, is an industrialized city in the northeastern region of Brazil. It is located in a metropolitan region that includes 22 other municipalities, and has *per capita* income of approximately US$ 110 and Human Development Index (HDI) of 0.72.

A total of 11,410 medical records regarding general hospital care were evaluated. To compose the sample, cases of people who presented facial trauma and who were treated with need for hospitalization were included. Exclusion criteria were: records that were considered incomplete (lacking three or more information), illegible (even when, after consultation with physician or maxillofacial surgeon, the information in the medical record was not yet deciphered), resulting in 244 cases.

The variables studied were: age (in years), gender (female / male), type of etiological agent of the face trauma^10^ (traffic accident, interpersonal violence, falls, others such as work accident and accident during the practice of sports), type of facial trauma^11^ ^,^ ^12^ (soft tissue injury - laceration, bruising, hematoma; mandible fracture, maxilla fracture, zygomatic complex fracture, nasal fracture, nasal-orbital-ethmoidal fracture, frontal fracture, fracture in more than one facial bone), presence of associated trauma in other regions of the body (yes / no), type of treatment (surgical / non-surgical), quarter of care (first / second / third / fourth) and length of hospital stay (in days).

Initially, descriptive statistical analysis was performed, which corresponded to the calculation of the absolute and relative frequencies of categorical variables and to the calculation of the central tendency (mean and median) and dispersion measures (standard deviation, minimum value, maximum value and interquartile range) of continuous variables. Subsequently, Cluster Analysis was used to describe the victims’ profile. This is a multivariate, exploratory statistical analysis designed to allocate individuals with characteristics similar to each other in the same group (cluster), in order to identify profiles or trends that could go unnoticed if other techniques were used.^13^ The method chosen was the TwoStep Cluster. One of the advantages of this method is the possibility of manipulating categorical and continuous variables simultaneously and the automatic identification of the number of empirical clusters based on the Bayesian and Akaike information criteria, which are used in a joint and comparative way to indicate the empirically optimal solution.^14^

For the conformation of the clusters, variables that were able to define clusters capable of better guiding the implementation of prevention, management, assistance and rehabilitation strategies were used. Thus, variables related to the sociodemographic characteristics of patients, to the etiological agents of traumas, the nature of lesions, treatment and evolution were chosen. For the application of the method, the criterion of choice for the selection of the number of clusters was the Bayesian Information Criterion (BIC) and the distance measure used was the Log-likelihood. It is known that the denomination of clusters is a subjective process, but it was tried to standardize the description of clusters in such a way that they represented the most remarkable findings in data and could guide the reader in the understanding of the main characteristics demarcated by empirically obtained clusters. In order to identify the variables that most contributed to the external differentiation of clusters, the analysis of the difference of proportions (Pearson’s Chi-square or Fisher’s Exact Test) and the F-test (ANOVA) was used. The confidence interval considered was 95%. The organization of the database and all statistical analyses were performed using IBM SPSS software version 20.

This study was submitted to and approved by the Ethics Research Committee on Human Beings of the State University of Paraíba (CAAE protocol No. 33813.4.0000.5187) and followed the National and International Standards of Ethics in Research with Human Beings.

## RESULTS

The mean age of victims was 31.16 years (SD = 15.17 years, minimum value: 1 year, maximum value: 78 years) and median of 27 years. The mean length of hospital stay was 6.32 days (SD = 7.75 days, minimum value: 1 day, maximum value: 28 days) and median 5 days. Table 1 presents the absolute and relative frequencies of variables related to the sociodemographic characteristics of patients, etiology and characteristics of traumas, type of treatment and quarter of care. The majority of patients were male (n = 224; 91.8%), and the male/female proportion was 11.2: 1. The main etiological agent of facial trauma corresponded to traffic accidents (n = 55; 63.5%) and the most frequent type of facial trauma was zygomatic complex fracture (n = 71; 29.1%) followed by situations of fracture in more than one facial bone (n = 49; 20.1%). In addition, it was observed that the presence of associated trauma in other regions of the body occurred in 16.4% of cases (n = 40), the type of treatment most adopted was surgical (n = 220; 90.2%), in the fourth quarter (n = 109; 44.7%), followed by the third quarter (n = 77; 31.6%).

**Table 1:** Absolute and relative frequencies of variables related to the sociodemographic characteristics of patients, etiology and characteristics of traumas, type of treatment and quarter of care.

Variables	n	%
Gender		
Female	20	8.2
Male	224	91.8
**Etiology**		
Traffic accident	155	63.5
Interpersonal violence	32	13.1
Falls	19	7.8
Others	38	15.6
**Facial trauma**		
Soft tissue injury	26	10.7
Mandible fracture	39	16.0
Maxilla fracture	15	6.1
Zygomatic complex fracture	71	29.1
Nasal fracture	44	18.0
Fracture in more than one facial bone	49	20.1
**Presence of associated trauma**		
Yes	40	16.4
No	204	83.6
**Type of treatment**		
Surgical	220	90.2
Non-surgical	24	9.8
**Quarter of care**		
First	23	9.4
Second	35	14.3
Third	77	31.6
Fourth	109	44.7

**Figure 1:**
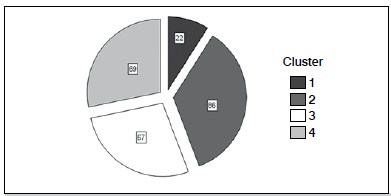
Absolute distribution of clusters.

Figure 1 shows the absolute distribution of clusters. The number of patients allocated to clusters 1, 2, 3 and 4 were, respectively, 22, 86, 67 and 69. Table 2 shows the distribution of clusters according to patient’s age, length of hospital stay, gender, etiology of facial trauma, type of facial trauma, presence of associated trauma in another region of the body, type of treatment and quarter of care. The variables selected for conformation of clusters that most contributed to the external differentiation among clusters were: length of hospital stay (p <0.001), etiology (p <0.001), type of facial trauma (p <0.001), associated trauma in another region of the body (p < 0.001), type of treatment adopted (p <0.001) and quarter of care (p <0.001). The automatic formation of four clusters with different profiles of patients was verified.

**Table 2:** Distribution of clusters according to patient's age, length of hospital stay, gender, etiology of facial trauma, type of facial trauma, presence of associated trauma in another region of the body, type of treatment and quarter of care.

Variables	1	2	3	4	Total	p-value
	(n = 22)	(n = 86)	(n = 67)	(n = 69)		
Mean age (standard deviation)	31.17 (±17.47)	33.23 (±17.15)	28.0 (±12.13)	31.67 (±14.22)	31.16 (15.17)	0.200
Mean length of hospital stay (standard deviation)	1.73 (±2.14)	4.35 (±3.91)	11.43 (±7.24)	5.28 (±2.85)	6.32 (5.75)	<0.001
**Gender**						**0.008**
Female	2 (9.1)	10 (11.6)	0 (0.0)	8 (11.6)	20 (8.2)	
Male	20 (90.9)	76 (88.4)	67 (100.0)	61 (88.4)	224 (91.8)	
**Etiology**						**<0.001**
Traffic accident	16 (72.7)	6 (7.0)	65 (97.0)	68 (98.6)	155 (63.5)	
Intepersonal violence	0 (0.0)	32 (37.2)	0 (0.0)	0 (0.0)	32 (13.1)	
Falls	0 (0.0)	17 (19.8)	1 (1.5)	1 (1.4)	19 (7.8)	
Others	6 (27.3)	31 (36.0)	1 (1.5)	0 (0.0)	38 (15.6)	
**Facial trauma**						**<0.001**
Soft tissue injury	17 (77.3)	9 (10.5)	0 (0.0)	0 (0.0)	26 (10.7)	
Mandible fracture	0 (0.0)	15 (17.4)	14 (20.9)	10 (14.5)	39 (16.0)	
Maxilla fracture	0 (0.0)	0 (0.0)	8 (11.9)	7 (10.1)	15 (6.1)	
Zygomatic complex fracture	3 (13.6)	21 (24.4)	30 (44.8)	17 (24.6)	71 (29.1)	
Nasal fracture	1 (4.5)	29 (33.7)	0 (0.0)	14 (20.3)	44 (18.0)	
Fracture in more than one facial bone	1 (4.5)	12 (14.0)	15 (22.4)	21 (30.4)	49 (20.1)	
**Presence of associated trauma**						**<0.001**
Yes	14 (63.6)	3 (3.5)	19 (28.4)	4 (5.8)	40 (16.4)	
No	8 (36.4)	83 (96.5)	48 (71.6)	65 (94.2)	204 (83.6)	
**Type of treatment**						**<0.001**
Surgical	1 (4.5)	83 (96.5)	67 (100.0)	69 (100.0)	220 (90.2)	
Non-surgical	21 (95.5)	3 (3.5)	0 (0.0)	0 (0.0)	24 (9.8)	
**Quarter of care**						**<0.001**
First	7 (31.8)	5 (5.8)	10 (14.9)	1 (1.4)	23 (9.4)	
Second	3 (13.6)	10 (11.6)	18 (26.9)	4 (5.8)	35 (14.3)	
Third	9 (40.9)	37 (43.0)	31 (46.3)	0 (0.0)	77 (31.6)	
Fourth	3 (13.6)	34 (39.5)	8 (11.9)	64 (92.8)	109 (44.7)	

Cluster 1 consisted essentially of patients with mean age of 31.17 years (SD = 17.47, minimum value = 1, maximum value = 68) and median of 28.5 years (IIQ = 18.8), males (n=20; 90.9%), traffic accident victims (n = 16; 72.7%), who presented facial trauma characterized by soft tissue injury (n = 17; 77.3%), associated trauma in other regions of the body (n = 14; 63.6%), treated in the third quarter (n = 9; 40.9%) and submitted to non-surgical treatment (n = 21, 95.5%), with mean length of hospital stay of 1.73 days (SD = 2.14); minimum value = 1; maximum value = 10 and median 1 day (IIQ = 2).

Cluster 2 consisted essentially of patients with mean age of 33.23 years (SD = 17.15, minimum value = 2, maximum value = 78) and median age of 31.5 years (IIQ = 23.0), males (n = 76; 88.4%), victims of interpersonal violence (n = 32; 37.2%) or other external causes (n = 31; 36.0%), who presented with nasal fracture (n = 29; 33.7%), had no associated trauma in other regions of the body (n = 83; 96.5%), were treated in the third quarter (n = 37; 43.0%) and submitted to surgical treatment (n= 83; 96.5%), with mean length of hospital stay of 4.35 days (SD = 3.91, minimum value = 1, maximum value = 18) and median three days (IIQ = 3).

Cluster 3 consisted essentially of patients with mean age of 28.0 years (SD = 12.13, minimum value = 12, maximum value = 69) and a median of 24 years (IIQ = 15.0), males (n = 67; 100.0%), traffic accident victims (n = 65; 97.0%), with zygomatic complex fracture (n = 30; 44.8%), no associated trauma in other regions of the body (n = 48; 71.6%), treated in the third quarter (n = 31; 46.3%) and submitted to surgical treatment (n = 67; 100.0%), with mean length of hospital stay of 11.43 days (SD = 7.24, minimum value = 1, maximum value = 28) and median of nine days (IIQ = 12).

Cluster 4 consisted essentially of patients with mean age of 31.67 (SD = 14.22, minimum value = 8, maximum value = 77), and median of 28 years (IIQ = 15.5), males (n = 61; 88.4%), with fracture in more than one facial bone (n = 21; 30.4%), no associated trauma in other regions of the body (n = 65; 94.2%), treated in the fourth quarter (n = 64; 92.8%), submitted to surgical treatment (n = 69; 100.0%), with mean length of hospital stay of 5.28 days (SD = 2.85, minimum value = 1, maximum value = 14) and median of five days (IIQ = 4).

## DISCUSSION

The high prevalence of facial trauma in males found in this study corroborates the results found by other authors,^5^ ^,^ ^15^ and this fact is probably attributed to the greater involvement of men in outdoor activities and their greater exposure to violent interactions. However, it is noteworthy that, due to the greater involvement of women in physical activity, high number of female drivers, as well as the increase in violence associated with greater participation of women in extra-community activities, together, contributes to their exposure to risk factors similar to those of men.^16^

Maxillofacial trauma was more frequent in young adult patients, in agreement with previous findings in literature.^17^ The frequent occurrence of these traumas at this stage of the life cycle can be attributed to the fact that this group performs exercises and dangerous sports; in addition to the use of transport means at high speeds.^18^ The greater victimization of young people is very worrying, since it may possibly generate sequels that could compromise their performance of work activities. Future studies should be carried out to assess the association between absenteeism and morbidity resulting from external causes, especially traffic accidents and interpersonal violence.

Of the four identified clusters, three were related to victims of traffic accidents, reflecting their prominent role as an etiological agent for facial trauma, especially fractures. This information corroborates previous studies in literature showing the high prevalence of traumas due to traffic accidents.^5^ ^,^ ^17^ Probably, due to high speed driving, non-permitted overtaking and the lack of citizenship exercise in traffic may explain the occurrence of traffic accidents in the region studied. Although not assessed in this study, alcohol consumption is an aspect to be considered in the etiology of facial fractures, and may be involved in traffic accidents. In many cases, patients attribute fracture to an accidental fall, omitting the alcohol consumption, which makes it difficult to verify the involvement of alcoholic beverages in cases of fractures.

The length of hospital stay is a crucial point that must be taken into account during the process of redesigning health services. In this study, the length of hospital stay ranged from 1 to 28 days. In the study developed by van Hout et al.,^19^ this period was much longer (1 to 127 days). An explanation for the longer hospitalization period would be the absence of a standard hospitalization time, as this varies according to the patient’s need. The most common fracture pattern in this study was that of the zygomatic complex, especially among patients in cluster 3, presenting a mean longer hospitalization time compared to those of the other clusters. The zygomatic region is commonly fractured due to its prominent anatomy on the face.^6^ ^,^ ^18^ With the exception of cluster 1, the type of treatment most adopted corresponded to the surgical one. This result is a reflection of the complexity of trauma cases. The greater the energy associated with the cause of trauma, the greater the trauma complexity and the greater the probability of surgical treatment.^20^

In general, the highest care frequency was recorded in the fourth quarter. In a 10-year study at the University Hospital of Innsbruck (Austria), Gassner et al.^11^ concluded that August was the month with the highest care frequency, emphasizing that this is a summer month in the north hemisphere. The distribution of months varies according to the place of study; and as Brazil is a tropical climate country, it has no drastic changes in temperature in seasons.^20^ A large popular festivity takes place in the region under study in June, which increases the number of people who come from neighboring cities and other states to celebrate the June celebrations. The lower number of treatments performed on the second quarter may be a reflection of the awareness campaigns for the prevention of accidents and violence events developed in recent years.

One of the limitations of this study is its cross-sectional design, not allowing establishing causal relationships, and the fact that the sample was of the intentional type. In addition, it was not possible to measure the impact of trauma on the quality of life of victims, which requires future investigations. Studies with appropriate methodology to evaluate the influence of the use of psychoactive substances and the occurrence of facial traumas are essential and represent an area that can be approached in future research. The results obtained are expected to substantially contribute for the planning of prevention and management actions in health, epidemiological surveillance and reorientation of assistance practices to victims of facial traumas due to external causes.

## CONCLUSION

According to the results obtained, it could be concluded that the majority of victims corresponded to men who were involved in traffic accidents, presenting fractures mainly of the zygomatic complex requiring surgical treatment.
